# Book Reviews

**Published:** 1907-01

**Authors:** 


### BOOK REVIEWS.

Atlas and Text Book of Dentistry, including Diseases of the Mouth, by Gustav Preiswerk, M. D., Ph. D., of the University of Basel. Authorized translation from the German. Edited by Geo. W. Warren, A. M., D. D. S., Professor of Principles and Practice of Operative Dentistry, Pennsylvania College of Dental Surgery. With 103 colored figures on 44 plates and 152 text illustrations. Three hundred and forty-three pages. Price, $3.50 net. Published by W. B. Saunders Co., Philadelphia and London. 1906.

The illustrations in this volume are the best we have ever noted in a volume of this character.

The colored plates are both exquisite and strikingly accurate and are alone worth the price of the volume.

The attempt to cover the entire subject of dentistry in a volume of this size is, we believe, a mistake, and the work naturally suffers from the limitations of such an attempt so far as the text is concerned. Taken altogether, however, it is a good book and well worth a careful study.

---

Dr. B—, a prominent dentist in Atlanta, Ga., in a personal letter to a dental friend recently said: "Not long ago I was called to see a man who had had an impacted wisdom tooth extracted by another dentist. Forty-eight hours after the extraction I saw the patient and found his jaws locked tightly together, and his face and neck so swollen that the chin and nipple were almost on a line. He was suffering intense pain.

"I immediately covered the swollen area with Antiphlogistine two inches thick, applied as hot as he could possibly stand. The dressing was changed every twelve hours for four days, when I was able to open the jaw.

"I found the cavity of the wisdom tooth had been packed with gauze. When I removed this foul smelling package, a stream of pus followed.

"I am indebted to Antiphlogistine for making an extremely difficult case end in excellent condition."

